# Bayesian^3^ Active Learning for the Gaussian Process Emulator Using Information Theory

**DOI:** 10.3390/e22080890

**Published:** 2020-08-13

**Authors:** Sergey Oladyshkin, Farid Mohammadi, Ilja Kroeker, Wolfgang Nowak

**Affiliations:** 1 Department of Stochastic Simulation and Safety Research for Hydrosystems, Institute for Modelling Hydraulic and Environmental Systems/SC SimTech, University of Stuttgart, Pfaffenwaldring 5a, 70569 Stuttgart, Germany; ilja.kroeker@iws.uni-stuttgart.de (I.K.); wolfgang.nowak@iws.uni-stuttgart.de (W.N.); 2 Department of Hydromechanics and Modelling of Hydrosystems, Institute for Modelling Hydraulic and Environmental Systems/SC SimTech, University of Stuttgart, Pfaffenwaldring 61, 70569 Stuttgart, Germany; farid.mohammadi@iws.uni-stuttgart.de

**Keywords:** machine learning, active learning, Gaussian process emulator, Bayesian inference, Bayesian model evidence, relative entropy, Kullback–Leibler divergence, information entropy

## Abstract

Gaussian process emulators (GPE) are a machine learning approach that replicates computational demanding models using training runs of that model. Constructing such a surrogate is very challenging and, in the context of Bayesian inference, the training runs should be well invested. The current paper offers a fully Bayesian view on GPEs for Bayesian inference accompanied by Bayesian active learning (BAL). We introduce three BAL strategies that adaptively identify training sets for the GPE using information-theoretic arguments. The first strategy relies on Bayesian model evidence that indicates the GPE’s quality of matching the measurement data, the second strategy is based on relative entropy that indicates the relative information gain for the GPE, and the third is founded on information entropy that indicates the missing information in the GPE. We illustrate the performance of our three strategies using analytical- and carbon-dioxide benchmarks. The paper shows evidence of convergence against a reference solution and demonstrates quantification of post-calibration uncertainty by comparing the introduced three strategies. We conclude that Bayesian model evidence-based and relative entropy-based strategies outperform the entropy-based strategy because the latter can be misleading during the BAL. The relative entropy-based strategy demonstrates superior performance to the Bayesian model evidence-based strategy.

## 1. Introduction

The greatest challenge of the scientific modeling workflow is to construct reliable and feasible models that can adequately describe underlying physical concepts and, at the same time, account for uncertainty [1]. Due to the computational complexity of the underlying physical concepts, numerical simulation models are often too expensive for applications tasks related to uncertainty quantification, risk assessment and stochastic model calibration. The great difficulty here is to establish a consistent and feasible framework that can provide appropriate conceptual descriptions and can simultaneously maintain a reliable time frame of simulations. The latter is the primary reason why a vast majority of ongoing research has been focusing on accelerating the forward model using surrogate models, such as response surfaces, emulators, meta-models, reduced-order models, etc. Due to the high computational costs of the original numerical simulation required for training runs of such surrogates, constructing surrogate models is still challenging. Classical machine learning approaches, such as artificial neural networks, require huge numbers of model evaluations.

A reasonably fast approach to quantify forward uncertainty has been established by Wiener [2], projecting a full-complexity model onto orthogonal polynomial bases over the parameter space. The conventional non-intrusive version of the polynomial chaos expansion [3,4] or its generalization towards data-driven descriptions [5,6] gained popularity during the last few decades because it can offer an efficient reduction of computational costs in uncertainty quantification [7,8,9]. Advanced extensions towards sparse quadrature [10], sparse integration rules [11,12,13], or multi-element polynomial chaos approaches [14,15] were applied to complex and computationally demanding applications.

Alternately to global [16] or local polynomial representation [17], other kernels functions have been widely used in applied mathematics [18] and machine learning [19]. Similar to polynomial chaos expansions, Gaussian process emulators (GPE), also known as Kriging for spatial prediction in the Geosciences [20], offer a linear representation through nonlinear kernels using fundamentals of probability theory. For that reason, GPEs are also known as Wiener–Kolmogorov prediction, after Norbert Wiener [2] and Andrey Kolmogorov [21]. GPEs also offer representation via various kernels, and have gained popularity for such machine learning tasks as classification [22] and regression problems [23]. A recent paper [24] compares various surrogate-based approaches using a common benchmark model for forward uncertainty quantification in carbon carbon dioxide storage.

Surrogate representation of the original physical model can be very helpful to accelerate forward modeling and assess prior uncertainty. Most versions of the surrogate methods named above need training runs of the original model to construct the surrogate. However, once additional information is available in the form of measurement data, then a reliable and feasible framework for inverse modeling is indispensable to account for the uncertainty that remains after model calibration. Bayesian inference [21] offers a rigorous stochastic framework for inverse modelling and for assessing the remaining uncertainty in model parameters and predictions [25]. Direct implementation of Bayesian principles for the original physical model is usually not feasible using Monte Carlo (MC) simulations [26] or even Markov chain Monte Carlo (MCMC) approaches [27]. Any advanced technique, such as thermodynamic integration [28], parallel tempering [29], nested sampling [30,31], subset simulation [32,33], or Gaussian mixture importance sampling [34] is still not feasible for applications where the original model is very expensive.

A recent trend toward stochastic calibration based on surrogate models offers iterative improvement of surrogate representations [35,36,37,38,39] within a limited simulation time budget. For example, the link between Bayesian inference and information theory introduced in [40] can help localize adaptively the relevant spots in the parameter space for surrogate training, according to information-theoretic arguments. Such a procedure of active learning should be very informative regarding the available observation data. It has the potential to adaptively improve the surrogate model in those regions of the parameter space that are most important for Bayesian inference, while including relevant information in an iterative manner.

To improve this procedure, the current paper will make use of the information theory [41,42,43,44], which is strongly linked to classical probability theory [21], information entropy [42] and cross entropy [41,45]. The latter have been widely used to measure expected uncertainty and information [46,47]. Relative entropy, also known as Kullback–Leibler divergence [43], quantifies the difference between two probability distributions. All aforementioned entropies have been widely used for model selection [48,49,50], optimal design of experiments [51,52,53,54] and machine learning [55,56,57]. A review on entropy, information theory, information entropy and Bayesian inference can be found in [58].

The key idea of GPEs is based on the assumption, that the model in the yet unexplored regions of the parameter space can be considered as a Gaussian process. Thus, besides application in geoscience, active learning for GPEs is also widely applied in computer sciences [59,60,61]. A comprehensive introduction into Gaussian processes and GPEs is provided in [62]. The idea of GPE-based surrogates was introduced in the context of Bayesian calibration of computer models [63] and extended for optimal selection of training points in [64,65] and extended with active learning concepts [13,38] by several authors. Since GPEs are described by their mean and covariance, the choice of a parametric covariance function and estimation of the related hyper-parameters is decisive for constructing the GPE. There are various works available in the literature that are focusing on estimation of the related hyper parameters. Usually, there are several covariance functions known and used in literature, but in particular the squared exponential covariance and the Matérn covariance functions play an important role in geophysical applications [66,67,68].

GPE surrogates have often been used to replicate computationally demanding models. Employing various learning function for selecting GPE training points helps to assure the accuracy in that procedure (see [69,70,71]). Conventionally, learning approaches only focus on GPE training for an underlying model without considering measurement data in the context of Bayesian updating of model parameters. Contrary to that, the study [72] makes use of data only (no numerical model involved) and constructs a GPE model that directly represents the underlying phenomena. It employs information entropy to perform an optimal design of experiments for sensor placements and, in doing so, it exploits the multivariate Gaussian distribution of the GPE model. The study [73] also looks at model-based optimal design of experiments for sensor/measurement selection. Specifically, they look at sample placement for contaminant source identification problems, where the contaminant source geometry is covered by model parameters that are to be inferred. Within their optimization, they used a GPE-based MCMC simulation with local GPE refinement as an auxiliary tool. This means that their active learning strategy is made for planning real-world data collection, not for planning model runs during GPE training. Recently, the study [74] constructed a framework for accelerating MCMCs in Bayesian inference of model parameters. They introduced local approximations of these models into the Metropolis–Hastings kernel of MCMC. In such a setting, the greatest challenge is to replicate behaviors of the original numerical model while accounting for the available observation data at acceptable computational costs. Going to that same direction, the work [75] uses entropy as a learning function in the context of Bayesian assimilation of available data, but it approximates the posterior distribution of model parameters as log normal and relies on a multivariate Gaussian approximation of entropy. Similarly, learning functions for GPE training in Bayesian parameter inference can focus on the posterior mean and variance of model parameters obtained via assimilation of available measurement data. Following this route, the paper [76] suggested minimizing the mean-squared (averaged over the parameter posterior distribution) predictive error of the GPE. Alternatively, the study [77] suggested minimizing the integrated posterior variance of the GPE, again involving an average over the parameter posterior distribution. However, the posterior distribution resulting from Bayesian assimilation of measurement data is typically not multivariate Gaussian and, hence, any corresponding assumptions should be avoided whenever possible. Therefore, the current paper avoids such unnecessary assumptions. Instead, it offers a fully Bayesian view that relies on integral quantities such as BME, RE and IE.

Moreover, the accuracy of GPE depends strongly on the selection of training points [13], and GPEs with different training points can be seen as different surrogate models. Therefore, the main challenge of GPE-based surrogates in Bayesain inference consists of selecting training runs to capture the relevant features of the underlying full-complexity physical model, while focusing its accuracy of the regions of a good fit with the available observation data.

The current paper introduces a novel GPE-based machine learning framework to replicate behaviors of a computational demanding physical model using training runs of that model. The suggested framework focuses the training runs optimally for the parameter inference from observation data in a fully Bayesian view. The introduced framework makes use of Bayesian theory on the three different levels: the first construction of GPE from available training runs and identification of hyper parameters usually employs classical Bayesian principles; the second, incorporation of the available observation data, could be rigorously acceded via Bayesian updating; the third, training runs should be well identified using an adaptive Bayesian active learning strategy for GPE construction that captures the relevant features of the full-complexity model and honor the available observation data. Therefore, the overall framework can be seen as fully Bayesian (i.e., Bayesian^3^) active learning or, in short, denoted as BAL in the paper. The novelty over the previous studies and our main focus lies in the theirs level where we suggest three novel active learning strategies incorporating the information theory.

The rest of the paper is structured as follows: Section 2 explores the connection between Bayesian inference and information theory for Gaussian process emulators. This section also emphasizes that information entropy and relative entropy for Bayesian parameter inference and for Bayesian active learning can be computed avoiding any assumption or unnecessary multidimensional integration. Section 3 summarises fundamental properties of Gaussian process emulators and offers the three novel Bayesian active learning strategies based on GPEs and information theory for Bayesian parameter inference. Section 4 demonstrates application of the suggested GPE-based BAL strategies using an analytical example and a carbon dioxide benchmark problem. Additionally, Section 4 shows evidence of convergence against a brute-force reference solution for all proposed BAL strategies and demonstrates parameter inference for the proposed active learning strategies.

## 2. Bayesian Inference with Information Theory for a Gaussian Process Emulator

### 2.1. Construction and Training of Gaussian Process Emulators

We will consider a full-complexity model M producing model response M(ω,x,y,z,t) that depends on the some multi-dimensional parameter input ω at each physical point in space (x,y,z) and time *t*. The uncertain modelling parameters ω form a vector of random variables $\omega =\left\{\omega_{1},\ldots ,\omega_{n}\right\}$ from the parameter space Ω, where *n* is the number of uncertain parameters.

A Gaussian process emulator S(ω,x,y,z,t) (i.e., surrogate model) provides an approximation of the full-complexity model M(ω,x,y,z,t) over the parameter space Ω and for each point of space (x,y,z) and time *t*:

(1) $$M\left(\omega ,x,y,z,t\right)\approx S\left(\omega ,x,y,z,t\right)=\underset{\mathrm{trend}}{\underset{︸}{\sum_{l=1}^{m}\beta_{l}\left(x,y,z,t\right)h_{l}\left(\omega \right)}}+\underset{\text{zero-mean GPE}}{\underset{︸}{u(\omega ,x,y,z,t)}}.$$

Here, $h_{l}\left(\omega \right)$ for l=1,…,m denote trend basis functions over the parameter space Ω, $h_{1}$ is a constant and $\beta_{l}\left(x,y,z,t\right)$ are unknown coefficients of the expansion that only depend on space (x,y,z) and time *t*. The last term u(ω,x,y,z,t) in Equation (1) indicates the Gaussian process (GP). To introduce this term, we will assume *u* to be a GP with zero mean E(u)=0 and covariance $Cov(\omega ,\omega^{\prime })$ between u(ω,x,y,z,t) and $u(\omega^{\prime },x,y,z,t)$ given by:

(2) $$Cov\left(\omega ,\omega^{\prime }\right)=E\left[u\left(\omega ,x,y,z,t\right)u\left(\omega^{\prime },x,y,z,t\right)\right]=k\left(\omega ,\omega^{\prime }\right).$$

Therefore, the GP term u(ω,x,y,z,t) is assumed to be Gaussian distributed $u\left(\omega ,x,y,z,t\right)∼N(0,k\left(\omega ,\omega^{\prime }\right))$ according to the covariance kernel function $k(\omega ,\omega^{\prime })$ for each point of space (x,y,z) and time *t*. It is worth mentioning that there are different choices of the covariance kernel function k(·,·) available. The most common ones are the squared exponential kernel $k_{\mathrm{SE}}\left(\cdot ,\cdot \right)$ (the same as the Gaussian kernel) and the Matérn kernel $k_{\mathrm{Mat}é\mathrm{rn},\nu }\left(\cdot ,\cdot \right)$, which are defined as follows:

(3) $$\begin{array}{cc} k_{\mathrm{SE}}\left(\omega ,\omega^{\prime }\right) & :=\sigma^{2}\exp\left(-\frac{1}{2}\sum_{j=1}^{n}\frac{{\left(\omega_{j}-\omega_{j}^{\prime }\right)}^{2}}{\lambda_{j}^{2}}\right),\\ k_{\mathrm{Mat}é\mathrm{rn},\nu }\left(\omega ,\omega^{\prime }\right) & :=\sigma^{2}\frac{2^{1-\nu }}{\Gamma (\nu )}\cdot {\left(\frac{\sqrt{2\nu }{∥\omega -\omega^{\prime }∥}_{2}}{\lambda }\right)}^{\nu }K_{\nu }\left(\frac{\sqrt{2\nu }{∥\omega -\omega^{\prime }∥}_{2}}{\lambda }\right). \end{array}$$

Here, $\lambda_{j}\equiv \lambda_{j}\left(x,y,z,t\right)$ and λ≡λ(x,y,z,t) represent the length scale of auto-correlation along each component $\omega_{j}$ with j=1,…,n, the variance parameter is denoted by $\sigma^{2}\equiv \sigma^{2}\left(x,y,z,t\right)$ and the gamma function is denoted by Γ. $K_{\nu }$ is the modified Bessel function of the second kind with the smoothness parameter ν≡ν(x,y,z,t). For a more comprehensive description of several kernel functions, we refer the reader to [62]. The parameters $\sigma^{2},\lambda ,\lambda_{j}$ and ν as well as the trend coefficients $\beta_{l}$ define the mean and correlation function, and are called hyper parameters of S.

Constructing a Gaussian process emulator S(ω,x,y,z,t) for the full-complexity physical model M(ω,x,y,z,t) in Equation (1) is based on training runs of the full model. Let us denote the training input parameters (training points) by $\omega_{T}={\left\{\omega_{T1},\ldots ,\omega_{TN_{T}}\right\}}^{T}$ and the corresponding model responses by $M_{T}={\left\{M_{T1},\ldots ,M_{TN_{T}}\right\}}^{T}$, where $N_{T}$ is a value greater than zero representing the number of training points corresponding to the number of full-model evaluations. Thus, the data set $\left\{\left(\omega_{Ti},M_{Ti}\right),i=1,\ldots ,N_{T}\right\}$ is the complete training set for the GPE. Here, and in the following, we drop the coordinates for space (x,y,z) and time *t* in our notation for the sake of readability.

First, we look at hyperparameter inference. According to the GP, we will assume that each instance of the model response $M_{Ti}$ for $i=1,\ldots ,N_{T}$ can be modeled in a probabilistic sense as:

(4) $$P\left(M_{Ti}|u\left(\omega_{Ti}\right),\;\omega_{Ti}\right)∼N\left(M_{Ti}|h{\left(\omega_{Ti}\right)}^{T}\beta +u\left(\omega \right),\sigma^{2}\right).$$

Introducing vector notation for $H={\left\{h\left(\omega_{T1}\right)\;\ldots h\left(\omega_{TN_{T}}\right)\right\}}^{T}$, $U=\{u{\left(\omega_{T1},\ldots ,\omega_{TN_{T}}\right\}}^{T}$, we can rewrite the GPE representation of a given parameter set ω in the following form:

(5) $$P\left(S|U\right)∼N\left(H\beta +U,\sigma^{2}\right).$$

Furthermore, the joint distribution of the random vector *U* for a given parameter set ω is given by:

(6) $$P\left(U|\omega \right)∼N(0,K\left(\omega ,\omega^{\prime }\right)),$$

where the (co)variance matrix $K(\omega ,\omega^{\prime })$ for parameter sets $\omega ,\omega^{\prime }$ is defined according to the covariance kernel functions as follows:

(7) $$K\left(\omega ,\omega^{\prime }\right)=\left(\begin{array}{ccc} k(\omega_{1},\omega_{1}^{\prime }) & \ldots & k(\omega_{1},\omega_{N_{T}}^{\prime })\\ \vdots & \ddots & \vdots \\ k(\omega_{N_{T}},\omega_{1}^{\prime }) & \ldots & k(\omega_{N_{T}},\omega_{N_{T}}^{\prime }) \end{array}\right).$$

Equations (5)–(7) allow for estimating the GPEs hyper parameters using several GP-based methods and concepts that are available in the literature [13,62,65], such as the maximum likelihood method or more advanced Bayesian principles [62]. The mentioned Bayesian updating that identifies hyper parameters of the GPE representation is well-known in the literature [13,62,65] and will not be addressed in the current paper. Bayesian principles are again employed to train the Gaussian process emulator S(ω,x,y,z,t) of the full-complexity physical model M(ω,x,y,z,t) based on the available training points $\omega_{T}={\left\{\omega_{T1},\ldots ,\omega_{TN_{T}}\right\}}^{T}$ and the corresponding model responses $M_{T}={\left\{M_{T1},\ldots ,M_{TN_{T}}\right\}}^{T}$ (see e.g., [78]). The training procedure provides the posterior multivariate Gaussian distribution $N_{\omega }\left(\mu_{S},\sigma_{S}\right)$ with a mean value $\mu_{S}$ and a standard deviation $\sigma_{S}$ of S(ω,x,y,z,t) for any given parameter set ω from the parameter space Ω. The accuracy of the GPE strongly depends on the number of training points and how they have been selected [13]. The question of how to select the training points properly is extremely relevant in general. When is even more challenging, but observation data should be incorporated into the full-complexity model via Bayesian inference of model parameters ω. Moreover, GPE with different training points can be seen as and indeed are different models. Therefore, the current paper will introduce a fully Bayesian view (Bayesian inference in Section 2.2 and Bayesian active learning in Section 3) on the construction of the GPE-based surrogate S(ω,x,y,z,t) that must capture the main features of the full-complexity model M(ω,x,y,z,t) and will be used to assist in Bayesian parameter inference.

### 2.2. Bayesian Updating on Observation Data Using GPE

Bayesian theory offers a statistically rigorous approach to deal with uncertainty during inference, providing probabilistic information on the remaining uncertainty in parameters and predictions while incorporating the available observation data D (vector $N_{D}\times 1$ with $N_{D}$ length of the observation data set) that is usually attributed at specific point in space (x,y,z) and time *t*. In the Bayesian framework, initial knowledge on modelling parameters ω is encoded in a prior probability distribution p(ω). After Bayesian parameter inference, one obtains a posterior probability distribution of the parameters p(ω|D), which is more informative than the prior distribution. Posterior probability distribution of the parameters p(S|D) could be obtained with the help of the full-complexity model M (i.e., p(ω|D,M)) or with the help of the surrogate model S (i.e p(ω|D,S)) according to the approximation in Equation (1). Due to the high computational demand of the original full complexity model, we will employ the last option in the current paper, i.e., p(ω|D))≡p(ω|D,S)≈p(ω|D,M)) (see more details in [79]).

Formally, the posterior parameter distribution p(ω|D) of *n* uncertain parameters forming the vector of random variables $\omega =\left\{\omega_{1},\ldots ,\omega_{n}\right\}$ is obtained by updating the prior parameter distribution p(ω) in the light of observed data D according to Bayes’ Theorem [21]:

(8) $$p\left(\omega |D\right)=\frac{p(D|S)p(\omega )}{p(D)},$$

where the term p(D|ω) is the likelihood function that quantifies how well the surrogate model’s predictions S(ω,x,y,z,t) match the observed data D (the full notation corresponding to p(D|ω,S) will be avoided in the paper). The term p(D) (i.e., p(D,S) in full notation) is called Bayesian model evidence (BME) and can be seen as a normalizing constant for the posterior distribution of the parameters ω.

In order to describe how well the GPE predictions S(ω,x,y,z,t) in physical space $\left\{x,y,z,t\right\}$ match the observed data D, we use the following likelihood function p(D|ω) assuming independent and Gaussian distributed measurement errors:

(9) $$p\left(D|\omega \right)={\left(2\pi \right)}^{-N_{D}/2}{\left|R\right|}^{-\frac{1}{2}}\exp\left[-\frac{1}{2}{\left(D-S(\omega ,x,y,z,t)\right)}^{T}R^{-1}\left(D-S(\omega ,x,y,z,t)\right)\right],$$

where R ($N_{D}\times N_{D}$) is the diagonal (co)variance matrix of measurement error ϵ.

The performance of Bayesian updating on available observation date using GPE surrogate can be assessed by employing BME, relative entropy and information entropy [80] that are introduced in the upcoming Section 2.3, Section 2.4 and Section 2.5.

### 2.3. Bayesian Model Evidence

The BME value p(D) in the denominator of Equation (8) indicates the quality of the model against the available data and can be obtained by integrating the Equation (8) over the parameter space Ω as:

(10) $$\mathrm{BME}\equiv p\left(D\right)=\int_{\Omega }p\left(D|\omega \right)p\left(\omega \right)d\omega ,$$

or

(11) $$\mathrm{BME}=E_{p(\omega )}\left(p(D|\omega )\right).$$

Therefore, BME p(D) can be directly estimated [81] from Equation (11) using Monte Carlo sampling techniques [82] on the GPE:

(12) $$E_{p(\omega )}\left(p(D|\omega )\right)\approx \frac{1}{N}\sum_{i=1}^{N}\left(p(D|\omega_{i})\right),$$

where *N* is the size of Monte Carlo sample.

We remark that the GPE-based surrogate model S(ω,x,y,z,t) contains approximation errors because the GPE is merely a surrogate model of the full-complexity model M(ω,x,y,z,t). Therefore, GPE-based BME value p(D,S) in Equation (11) is an approximation of full-complexity model BME value p(D,M), i.e., $\mathrm{BME}=E_{p(\omega )}\left(p(D|\omega ),S\right)\approx p\left(\omega |D,M\right))$. A correction factor for BME value can be incorporated similar to the one in [79].

### 2.4. Relative Entropy

Relative entropy (RE), also called Kullback–Leibler divergence, measures the difference between two probability distributions [43] in the Bayesian context. Relative entropy $D_{\mathrm{KL}}\left[p(\omega |D),p(\omega )\right]$ measures the so-called information geometry in moving from the prior p(ω) to the posterior p(ω|D), or the information lost when p(ω) is used to approximate p(ω|D):

(13) $$D_{\mathrm{KL}}\left[p(\omega |D),p(\omega )\right]=\int_{\Omega }\ln\left[\frac{p(\omega |D)}{p(\omega )}\right]p\left(\omega |D\right)d\omega ,$$

Estimating the relative entropy in Equation (13) usually requires a multidimensional integration that is often infeasible for most applied problems. However, employing Equation (13) and definition (5) from the recent findings in the paper [40], we avoid this multidimensional integration:

(14) $$D_{\mathrm{KL}}\left[p(\omega |D),p(\omega )\right]=-\ln\mathrm{BME}+\int_{\Omega }\ln\left[p(D|\omega )\right]p\left(\omega |D\right)d\omega .$$

Therefore, relative entropy $D_{\mathrm{KL}}\left[p(\omega |D),p(\omega )\right]$ can be directly estimated from Equation (14) using Monte Carlo sampling techniques on the GPE:

(15) $$D_{\mathrm{KL}}\left[p(\omega |D),p(\omega )\right]=-\ln\mathrm{BME}+E_{p(\omega |D)}\left(\ln\left[p(D|\omega )\right]\right).$$

The expression for relative entropy in Equation (15) employs the prior-based estimation of BME values in Equation (11) and a posterior-based expectation of the likelihood $E_{p(\omega |D)}\left(\ln\left[p(D|\omega )\right]\right)$ that could be obtained, e.g., using a rejection sampling technique or similar [26] as:

(16) $$E_{p(\omega |D)}\left(\ln\left[p(D|\omega )\right]\right)\approx \frac{1}{N_{p}}\sum_{i=1}^{N_{p}}\left(\ln\left[p(D|\omega_{i})\right]\right),$$

where $N_{p}$ is the size of posterior sample according to rejection sampling.

### 2.5. Information Entropy

Information entropy (IE) is a measure of the expected missing information and can also be seen as uncertainty of a random variable ω. According to Shannon [42], the information entropy $H\left[p(\omega |D)\right]$ for a random variable ω with (posterior) parameter distribution p(ω|D) is defined as:

(17) $$H\left[p(\omega |D)\right]=-\int_{\Omega }\ln\left[p(\omega |D)\right]p\left(\omega |D\right)d\omega .$$

However, information entropy $H\left[p(\omega |D)\right]$ in Equation (17) can not be computed directly from a posterior sample because the posterior density values p(ω|D) are unknown. To overcome this situation, we will employ the definition of $D_{\mathrm{KL}}\left[p(\omega |D),p(\omega )\right]$ in Equation (13) to express the information entropy as:

(18) $$H\left[p(\omega |D)\right]=-\int_{\Omega }\ln\left[p(\omega )\right]p\left(\omega |D\right)d\omega -D_{\mathrm{KL}}\left[p(\omega |D),p(\omega )\right].$$

Therefore, employing Equation (15), information entropy can be directly estimated according to Equation (A3) in the paper [40] using Monte Carlo sampling techniques on the GPE:

(19) $$H\left[p(\omega |D)\right]=\ln\mathrm{BME}-E_{p(\omega |D)}\left(\ln\left[p(\omega )\right]\right)-E_{p(\omega |D)}\left(\ln\left[p(D|\omega )\right]\right).$$

Equation (19) does not contain any assumptions and avoids all multidimensional density estimations and integrals in Equation (17). It employs the prior-based estimation of BME values in Equation (10) and a posterior-based expectation of prior densities $E_{p(\omega |D)}\left(\ln\left[p(\omega )\right]\right)$ and likelihoods $E_{p(\omega |D)}\left(\ln\left[p(D|\omega )\right]\right)$. The posterior-based expectation of prior log-densities could be obtained as well using rejecting sampling techniques [26]:

(20) $$E_{p(\omega |D)}\left(\ln\left[p(\omega )\right]\right)\approx \frac{1}{N_{p}}\sum_{i=1}^{N_{p}}\left(\ln\left[p(\omega_{i})\right]\right).$$

## 3. Bayesian Active Learning for Gaussian Process Emulators in Parameter Inference

Section 2 described the standard construction of GPE S(ω,x,y,z,t) based on available training runs of the physical model $M_{T}={\left\{M_{T1},\ldots ,M_{TN_{T}}\right\}}^{T}$. As we want to infer the model parameters of the underlying physical model assisted by the constructed GPE surrogate, the training runs of the full model must ensure appropriate convergence. Specifically, such the GPE surrogate S(ω,x,y,z,t) must captures the main global features of the full-complexity model M(ω,x,y,z,t) and, at the same time, have local accuracy in the region of high posterior density p(ω|D) that will emanate during Bayesian inference. However, these regions for local accuracy are unknown a priori. Therefore, the current Section 3 focuses on iterative selection of training points. It employs the link between Bayesian inference and information theory [40] similar to Section 2 in order to perform Bayesian active learning (BAL). The later will iteratively select new training point as the regions with required local accuracy becomes progressively clear during the Bayesian updating of a Gaussian process emulator described in Section 2.

We will consider that the GPE surrogate S(ω,x,y,z,t) in Equation (1) has been constructed based on at least one training point ($N_{T}\geq 1$) in the parameter space $\omega_{T}={\left\{\omega_{T1},\ldots ,\omega_{TN_{T}}\right\}}^{T}$ using the corresponding model responses $M_{T}={\left\{M_{T1},\ldots ,M_{TN_{T}}\right\}}^{T}$. The goal of the current Section 3 is to identify the next training point $\omega_{T}^{BAL}$ that should be incorporated into the GPE surrogate S(ω,x,y,z,t). To do so, we will introduce three Bayesian active learning strategies that are based on Bayesian model evidence (Section 3.2), relative entropy (Section 3.3), and information entropy (Section 3.4). These strategies avoid unnecessary approximations and assumptions (such as maximum likelihood estimation, multivariate Gaussian posterior, etc.). Once a new training point $\omega_{T}^{BAL}$ has been identified, the model should be evaluated in that new point and the GPE in Equation (1) should be updated with typical GPE-inherent methods. In that way, the presented GPE-based fully Bayesian approach could help to calibrate the physical model to the available measurement data at the reduced computational costs. However, the GPE representation could never be better than the underlying physical model.

### 3.1. Bayesian Inference of Gaussian Process Emulator Incorporating Observation Data

The GPE is a collection of random functions over the parameter space Ω, i.e., a random model response S(ω,x,y,z,t) for each point of space (x,y,z) and time *t*. The Bayesian identification of hyper parameters during the training on the available model runs $M_{T}\left(\omega ,x,y,z,t\right)$ in Section 2.1 provides the multivariate Gaussian distribution $N_{\omega }\left(\mu_{S},\sigma_{S}\right)$ of the model response S(ω,x,y,z,t) forming response space S for any given parameter set ω from the parameter space Ω. Here, $\mu_{S}$ is a mean value and $\sigma_{S}$ is a standard deviation of model response S(ω,x,y,z,t) at each point of space (x,y,z) and time *t*. Therefore, we can explore the parameter space Ω using the exploration parameter set $\omega_{E}$ and we can assess how the obtained multivariate Gaussian distribution $N_{\omega_{E}}\left(\mu_{S},\sigma_{S}\right)$ meet the observation data D. According to the Bayesian framework [21], we can obtain a posterior probability distribution $p_{\omega_{E}}^{BAL}\left(S|D\right)$ of the model response for the given parameter set $\omega_{E}$, incorporating the observed data D:

(21) $$p_{\omega_{E}}^{BAL}\left(S|D\right)=\frac{p_{\omega_{E}}^{BAL}\left(D|S\right)N_{\omega_{E}}\left(\mu_{S},\sigma_{S}\right)}{p_{\omega_{E}}^{BAL}\left(D\right)},$$

where the term $p_{\omega_{E}}^{BAL}\left(D|S\right)$ is the likelihood function that quantifies how well the GPE predictions $S(\omega_{E},x,y,z,t)$ drawn from the multivariate Gaussian $N_{\omega_{E}}\left(\mu_{S},\sigma_{S}\right)$ match the observed data D and the term $p_{\omega_{E}}^{BAL}\left(D\right)$ is BME value of GPE for the given parameter set $\omega_{E}$.

Assuming independent and Gaussian distributed measurement errors, the likelihood function $p_{\omega_{E}}^{BAL}\left(D|S\right)$ can be written as:

(22) $$p_{\omega_{E}}^{BAL}\left(D|S\right)={\left(2\pi \right)}^{-N_{D}/2}{\left|R\right|}^{-\frac{1}{2}}\exp\left[-\frac{1}{2}{\left(D-S(\omega_{E},x,y,z,t)\right)}^{T}R^{-1}\left(D-S(\omega_{E},x,y,z,t)\right)\right],$$

where $S\left(\omega_{E},x,y,z,t\right)∼N_{\omega_{E}}\left(\mu_{S},\sigma_{S}\right)$.

### 3.2. Model Evidence-Based Bayesian Active Learning

As already mentioned in Section 2.2, BME is often used for model selection in order to identify the most suitable model among a set of competing models or to rank the competing models. During the active learning procedure, one has to identify the best “model” in the sense of the next trained version of GPE, i.e., the best position *d* of sampling point $\omega_{E}$. This point $\omega_{E}$ can be chosen such that it provides the highest BME of the next trained GPE. Therefore, BME value $p_{\omega_{E}}^{BAL}\left(D\right)$ for each point $\omega_{E}$ in the prior parameter space providing models responses S(ω,x,y,z,t) that forms a response space *Y* can be obtained using the following equation:

(23) $${\mathrm{BME}}^{BAL}\equiv p_{\omega_{E}}^{BAL}\left(D\right)=\int_{S}p_{\omega_{E}}^{BAL}\left(D|S\right)N_{\omega_{E}}\left(\mu_{S},\sigma_{S}\right)dS.$$

Equation (23) shows that ${\mathrm{BME}}^{BAL}$ is equal to the expected value $E_{N_{\omega_{E}}\left(\mu_{S},\sigma_{S}\right)}$ of the likelihood $p_{\omega_{E}}^{BAL}\left(D|S\right)$ over the prior $N_{\omega_{E}}\left(\mu_{S},\sigma_{S}\right)$ that GPE provides after training:

(24) $${\mathrm{BME}}^{BAL}=E_{N_{\omega_{E}}\left(\mu_{S},\sigma_{S}\right)}\left(p_{\omega_{E}}^{BAL}\left(D|S\right)\right).$$

The value ${\mathrm{BME}}^{BAL}$ can be directly estimated from Equation (24) using Monte Carlo sampling techniques [82] on the GPE:

(25) $$E_{N_{\omega_{E}}\left(\mu_{S},\sigma_{S}\right)}\left(p_{\omega_{E}}^{BAL}\left(D|S\right)\right)\approx \frac{1}{N^{BAL}}\sum_{i=1}^{N^{BAL}}\left(p_{\omega_{E_{i}}}^{BAL}\left(D|S\right)\right),$$

where $N^{BAL}$ is the size of Monte Carlo sample for Bayesian active learning.

Therefore, by formal maximization of the model evidence ${\mathrm{BME}}^{BAL}$, one can find the next training point $\omega_{T}^{BAL}$ from the parameter space Ω:

(26) $$\omega_{T}^{BAL}={arg max}_{\omega_{E}\in \Omega }{\mathrm{BME}}^{BAL}\left(\omega_{E}\right).$$

### 3.3. Relative Entropy-Based Bayesian Active Learning

Relative entropy is usually employed for Bayesian experimental design [51] to maximize the expected (marginalized) utility [53]. In the current paper, we will introduce the relative entropy $D_{\mathrm{KL}}^{\mathrm{BAL}}\left[p_{\omega_{E}}^{BAL}\left(S|D\right),N_{\omega_{E}}\left(\mu_{S},\sigma_{S}\right)\right]$ to assess the information geometry in moving the GPE from the multivariate Gaussian prior $N_{\omega_{E}}\left(\mu_{S},\sigma_{S}\right)$ to its posterior $p_{\omega_{E}}^{BAL}\left(S|D\right)$ during the active learning procedure. Formally, the relative entropy $D_{\mathrm{KL}}^{\mathrm{BAL}}\left[p_{\omega_{E}}^{BAL}\left(S|D\right),N_{\omega_{E}}\left(\mu_{S},\sigma_{S}\right)\right]$ can be defined for each sampling point $\omega_{E}$ from the parameter space Ω as following:

(27) $$D_{\mathrm{KL}}^{\mathrm{BAL}}\left[p_{\omega_{E}}^{BAL}\left(S|D\right),N_{\omega_{E}}\left(\mu_{S},\sigma_{S}\right)\right]=\int_{S}\ln\left[\frac{p_{\omega_{E}}^{BAL}\left(S|D\right)}{N_{\omega_{E}}\left(\mu_{S},\sigma_{S}\right)}\right]p_{\omega_{E}}^{BAL}\left(S|D\right)dS.$$

Similar to Section 2.4, we can avoid multidimensional integration in Equation (27) using Equation (13) and definition (5) from the paper [40]:

(28) $$D_{\mathrm{KL}}^{\mathrm{BAL}}\left[p_{\omega_{E}}^{BAL}\left(S|D\right),N_{\omega_{E}}\left(\mu_{S},\sigma_{S}\right)\right]=-\ln{\mathrm{BME}}^{BAL}+E_{p_{\omega_{E}}^{BAL}\left(S|D\right)}\left(\ln\left[p_{\omega_{E}}^{BAL}\left(D|S\right)\right]\right).$$

The posterior-based expectation of the log-likelihood could be obtained using a rejection sampling technique [26] on the GPE:

(29) $$E_{p_{\omega_{E}}^{BAL}\left(S|D\right)}\left(\ln\left[p_{\omega_{E}}^{BAL}\left(D|S\right)\right]\right)\approx \frac{1}{N_{p}^{BAL}}\sum_{i=1}^{N_{p}^{BAL}}\left(\ln\left[p_{\omega_{E_{i}}}^{BAL}\left(D|S\right)\right]\right),$$

where $N_{p}^{BAL}$ is the size of the posterior sample according to rejection sampling for Bayesian active learning.

Therefore, during the active learning procedure, we will identify the sampling point $\omega_{T}^{BAL}$ from the parameter space Ω that corresponds to maximum relative entropy $D_{\mathrm{KL}}^{\mathrm{BAL}}\left[p_{\omega_{E}}^{BAL}\left(S|D\right),N_{\omega_{E}}\left(\mu_{S},\sigma_{S}\right)\right]$:

(30) $$\omega_{T}^{BAL}={arg max}_{\omega_{E}\in \Omega }D_{\mathrm{KL}}^{\mathrm{BAL}}\left[p_{\omega_{E}}^{BAL}\left(S|D\right),N_{\omega_{E}}\left(\mu_{S},\sigma_{S}\right)\right].$$

It is evident that the optimization problem for RE value in Equation (30) relies not only on ${\mathrm{BME}}^{BAL}$ values from Equation (24). It also relies on the cross entropy represented by the term $E_{p_{\omega_{E}}^{BAL}\left(S|D\right)}\left(\ln\left[p_{\omega_{E}}^{BAL}\left(D|S\right)\right]\right)$ that reflects how likelihood informative for the posterior (see details in [40]). The last term could be obtained using a rejection sampling technique using the GPE evaluations.

### 3.4. Information Entropy-Based Bayesian Active Learning Criterion

Minimizing the expected information loss in terms of information entropy [42] has been suggested to identify the best fitting model [83] and is often used in machine learning. Again seeing the GPE with different training points as different models, we will introduce the information entropy $H^{BAL}\left[p_{\omega_{E}}^{BAL}\left(S|D\right)\right]$ to assess information loss for each parameter set $\omega_{E}$:

(31) $$H^{BAL}\left[p_{\omega_{E}}^{BAL}\left(S|D\right)\right]=-\int_{S}\ln\left[p_{\omega_{E}}^{BAL}\left(S|D\right)\right]p_{\omega_{E}}^{BAL}\left(S|D\right)dS.$$

Similar to Section 2.5, using Equation (A3) from the paper [40], the information entropy in Equation (31) can be written as follows:

(32) $$H^{BAL}\left[p_{\omega_{E}}^{BAL}\left(S|D\right)\right]=\ln{\mathrm{BME}}^{BAL}-E_{p_{\omega_{E}}^{BAL}\left(S|D\right)}\left(\ln\left[N_{\omega_{E}}\left(\mu_{S},\sigma_{S}\right)\right]\right)-E_{p_{\omega_{E}}^{BAL}\left(S|D\right)}\left(\ln\left[p_{\omega_{E}}^{BAL}\left(D|S\right)\right]\right),$$

where the posterior-based expectation $E_{p_{\omega_{E}}^{BAL}\left(S|D\right)}\left(\ln\left[N_{\omega_{E}}\left(\mu_{S},\sigma_{S}\right)\right]\right)$ could be obtained as well using a rejection sampling technique [26] on the GPE:

(33) $$E_{p_{\omega_{E}}^{BAL}\left(S|D\right)}\left(\ln\left[N_{\omega_{E}}\left(\mu_{S},\sigma_{S}\right)\right]\right)\approx \frac{1}{N_{p}^{BAL}}\sum_{i=1}^{N_{p}^{BAL}}\left(\ln\left[N_{\omega_{E_{i}}}\left(\mu_{S},\sigma_{S}\right)\right]\right),$$

All terms in Equation (32) could be obtained directly avoiding any multidimensional integration using prior-based or posterior-bases sampling from the GPE, such as rejecting sampling techniques. Therefore, to perform active learning, we will rely on the parameter set $\omega_{T}^{BAL}$ that corresponds to the minimum of information entropy $H^{BAL}\left[p_{\omega_{E}}^{BAL}\left(S|D\right)\right]$:

(34) $$\omega_{T}^{BAL}={arg min}_{\omega_{E}\in \Omega }H^{BAL}\left[p_{\omega_{E}}^{BAL}\left(S|D\right)\right].$$

Equation (34) that minimizes the IE value in Equation (32). It makes use of ${\mathrm{BME}}^{BAL}$ in Equation (24) and cross entropy $E_{p_{\omega_{E}}^{BAL}\left(S|D\right)}\left(\ln\left[p_{\omega_{E}}^{BAL}\left(D|S\right)\right]\right)$ similar to Equation (28). Moreover, Equation (32) shows that the information entropy $H^{BAL}\left[p_{\omega_{E}}^{BAL}\left(S|D\right)\right]$ relies on cross entropy represented and a posterior-based expectation of multivariate Gaussian prior $E_{p_{\omega_{E}}^{BAL}\left(S|D\right)}\left(\ln\left[N_{\omega_{E}}\left(\mu_{S},\sigma_{S}\right)\right]\right)$.

## 4. Application of GPE-Based Bayesian Active Learning

In the previous section, we have introduced three strategies for Bayesian^3^ active learning during the GPE-assisted Bayesian updating of model parameters as described in Section 2. The current section will make use of an analytical example in Section 4.1 and a carbon dioxide benchmark problem in Section 4.2 to illustrate the suggested active learning strategies from Section 3.

In the present work, we use the Matlab *fitrgp* function [78] to obtain the values of GPE parameters and hyper parameters introduced in Section 2.1 via a traditional Bayesian training on the available model runs. The proposed Bayesian active learning strategies in Section 3 for Bayesian inference in Section 2 have been implemented as an extension of the existing Matlab *fitrgp* function. The fully Bayesian^3^ active learning extension of *fitrgp* function is available online for the reader through Matlab file exchange [84]. For the sake of consistency, in the current publication, we have used the *fitrgp* function together with the squared exponential kernel $k_{\mathrm{SE}}\left(\cdot ,\cdot \right)$ as defined in Equation (3) for all examples. However, various kernel functions could be easily selected within Matlab *fitrgp* function using various training options. Therefore, the reader is invited to test the suggested Bayesian active learning strategies for own needs exploring the full range of Matlab *fitrgp* functionality.

### 4.1. Bayesian Active Learning for an Analytical Test Case

#### 4.1.1. Scenario Set up

We will consider a test case scenario in the form of a nonlinear analytical function M(ω,t) of ten (n=10) uncertain parameters $\omega =\left\{\omega_{1},\ldots ,\omega_{n}\right\}$ from the paper [40]:

(35) $$M\left(\omega ,t\right)={\left(\omega_{1}^{2}+\omega_{2}-1\right)}^{2}+\omega_{1}^{2}+0.1\omega_{1}\exp\left(\omega_{2}\right)-2\omega_{1}\sqrt{0.5t}+1+\sum_{i=3}^{n}\frac{\omega_{i}^{3}}{i}.$$

The uncertain parameters ω in Equation (35) are considered to be independent and uniformly distributed with $\omega_{i}∼U\left(-5,5\right)$ for $i=\bar{1,10}$. The prior assumptions on the parameters will be updated using synthetic observation data $D=M(\omega ,t_{k})$ with $t_{k}=\left(k-1\right)/9$ and $k=\bar{1,10}$ that correspond to the parameter set $\omega_{i}=0$∀i. The standard deviation of the measurement error is considered to be $\sigma_{D}=2$.

#### 4.1.2. Likelihood Reconstruction during Bayesian Active Learning

We will construct the Gaussian process emulator S(ω,t) in Equation (1) for the test case problem in Equation (35) to approximate the full model M(ω,t) in the parameter space ω for each point of time *t*. We will start the Bayesian active learning with one training point only ($N_{T}=1$). The starting training point corresponds to the mean value of the uncertain parameters ω, i.e., $\omega_{T}=E_{p(\omega )}\left(\omega \right)$. We will perform the Bayesian updating in Equation (8) using Monte Carlo sampling [26] on the constructed GPE surrogate S(ω,t) with sample size $N=10^{5}$ (alternative approaches can be used similarly). In order to identify the next training points for the GPE iteration, we employ the three Bayesian active learning strategies introduced in Section 3: the model evidence-based strategy in Equation (26), the relative entropy-based strategy in Equation (30) and the information entropy-based strategy in Equation (34).

**Figure 1:**
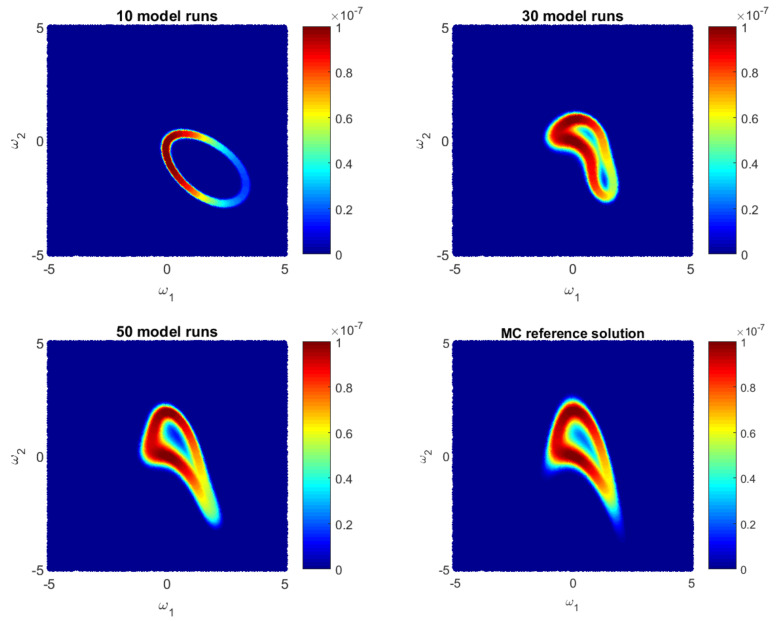
Likelihood values during Bayesian **BME-based** active learning as approximate by the Gaussian process emulator and by a reference Monte Carlo solution for a 2D reduction of the 10D problem.

**Figure 2:**
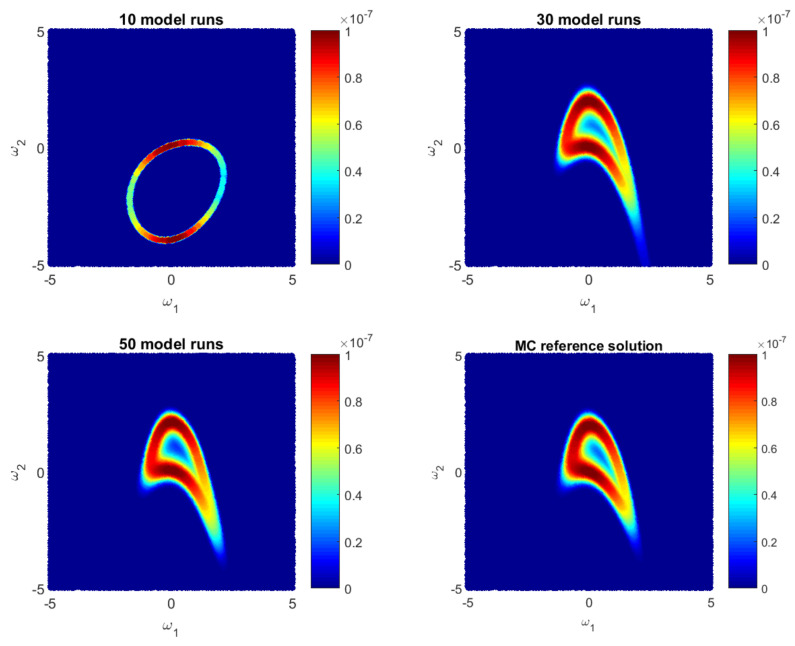
Likelihood values during Bayesian **Relative Enrtopy-based** active learning as approximate by the Gaussian process emulator and by a reference Monte Carlo solution for a 2D reduction of the 10D problem.

**Figure 3:**
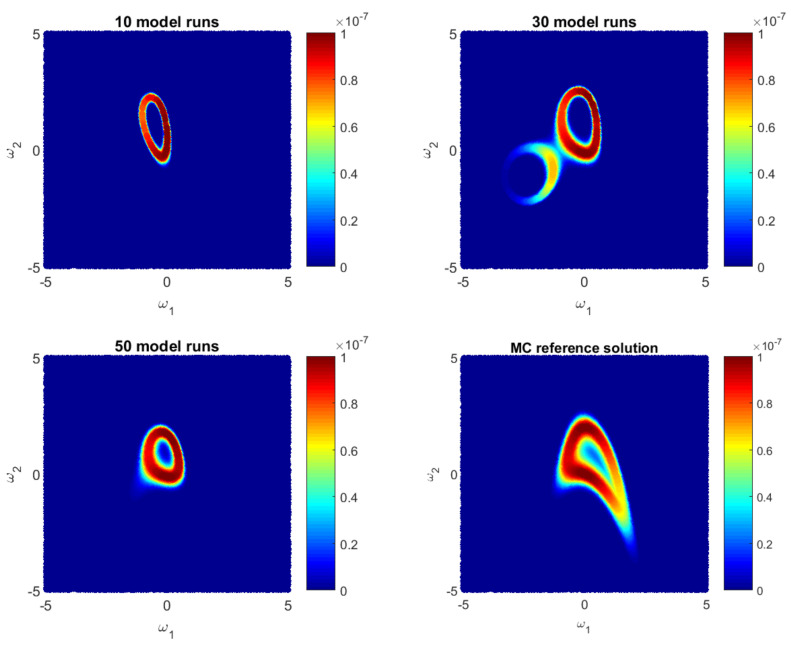
Likelihood values during Bayesian **Entropy-based** active learning as approximate by the Gaussian process emulator and by a reference Monte Carlo solution for a 2D reduction of the 10D problem.

Let us illustrate how the GPE-based likelihood function updates during the Bayesian active learning procedure. Additionally, we will assess the corresponding computational costs in terms of number of full model runs. For illustrative purposes, we will reduce the 10D problem (35) to a 2D problem with only two parameters, i.e., $\omega_{i}=0$ for $i=\bar{3,10}$. Figure 1, Figure 2 and Figure 3 show how the GPE’s likelihood function cover the 2D parameter space during active learning based on a BME-based strategy, RE-based strategy and IE-based strategy, respectively. Moreover, Figure 1, Figure 2 and Figure 3 show Monte Carlo reference solutions that have been obtained directly using Monte Carlo sampling on the original model, introduced in Section 4.1.1.

The RE-based Bayesian active learning captures the non-Gaussian aspects of the analysed problem in a remarkably effective manner in comparison to the BME-based and the IE-based approaches. The RE-based active learning provides a likelihood estimation that is practically identical to the MC reference solution after 25 model runs. The BME-based strategy captures the main features only in the beginning and requires a longer learning procedure to reflect details of the reference solution. The reason is that the RE relies on both BME and cross entropy that indicates how informative the likelihood for the posterior are (see Equation (28)). Contrary to the BME-based and the RE-based strategies, the IE-based active learning manages to cover only partially the likelihood function in the 2D parameter space for the given computational budget. Additionally, it shows a stagnation during the learning procedure, where 50-model-run training shows no significant improvement in comparison to 30-model-run training (see Figure 3). The difference between the RE and the IE-based active learning strategies consists in the second term in Equation (32). That term denotes the cross entropy and reflects how informative the trained multivariate Gaussian distribution $N_{\omega_{E}}\left(\mu_{S},\sigma_{S}\right)$ of GPE is, for the posterior $p_{\omega_{E}}^{BAL}\left(S|D\right)$. Formally, it can be seen as a posterior-based expectation of multivariate Gaussian distribution $E_{p_{\omega_{E}}^{BAL}\left(S|D\right)}\left(\ln\left[N_{\omega_{E}}\left(\mu_{S},\sigma_{S}\right)\right]\right)$ and it can overcome the RE value in Equation (32). Apparently, once the trained distribution $N_{\omega_{E}}\left(\mu_{S},\sigma_{S}\right)$ is extremely informative, then the IE-based active learning suggests to add new training point where $N_{\omega_{E}}\left(\mu_{S},\sigma_{S}\right)$ is already very similar to the posterior $p_{\omega_{E}}^{BAL}\left(S|D\right)$. Therefore, the last property could lead to a stagnation of the information entropy-based active learning, similar to Figure 3.

#### 4.1.3. Assessment of Information Arguments during Bayesian Active Learning

**Figure 4:**
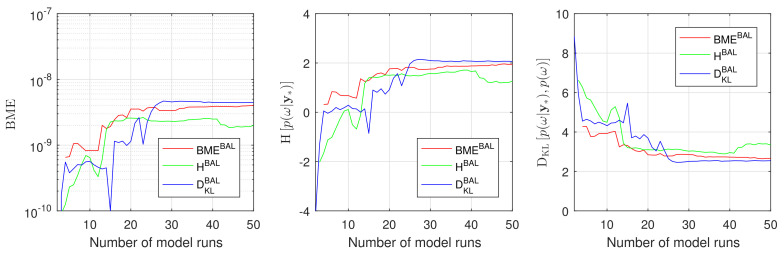
Bayesian model evidence, Information entropy and Relative entropy estimates during Bayesian active learning for Gaussian process emulator for a 2D reduction of the 10D problem: BME-based active learning (red line), IE-based active learning (green line) and RE-based active learning (blue line).

To assess the overall performance of the introduced Bayesian active learning strategies in Section 3, we compute Bayesian model evidence p(D), relative entropy $D_{\mathrm{KL}}\left[p(\omega |D),p(\omega )\right]$ and information entropy $H\left[p(\omega |D)\right]$. To do so, we will employ the resulting GPE surrogates in Equation (11), (15) and (19), correspondingly. In that way, we avoid any unnecessary multidimensional integration or density estimation via Monte Carlo integration. Figure 4 illustrates how the Bayesian model evidence, the information entropy and the relative entropy adjust their value during Bayesian active learning for the discussed 2D reduction of the original 10D problem. Figure 4 shows the results of the BME-based, the IE-based and the RE-based active learning using red, green and blue lines, respectively. All three approaches reach their plateaus after approximately 20–30 active learning steps that corresponds to 20–30 runs of the original model.

**Figure 5:**
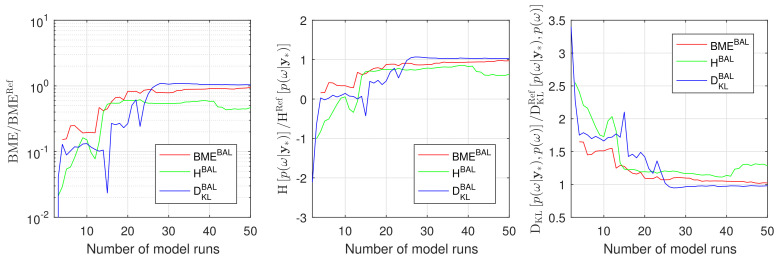
Convergence of Bayesian model evidence, Information entropy and Relative entropy estimates during Bayesian active learning for Gaussian process emulator to the reference Monte Carlo solution for a 2D reduction of the 10D problem: BME-based active learning (red line), IE-based active learning (green line) and RE-based active learning (blue line).

A proper conclusion, however, could be drawn once the obtained BME, $D_{\mathrm{KL}}\left[p(\omega |D),p(\omega )\right]$ and $H\left[p(\omega |D)\right]$ are compared against their reference solutions. Therefore, we compute all the reference values denoted here as ${\mathrm{BME}}^{\mathrm{Ref}}$, $D_{\mathrm{KL}}^{\mathrm{Ref}}\left[p(\omega |D),p(\omega )\right]$ and $H^{\mathrm{Ref}}\left[p(\omega |D)\right]$ employing the Equations (11), (15) and (19) avoiding any assumptions or density estimations. To do so, we evaluate the original model M instead of the surrogate S in the Equations (11), (15) and (19) for the available Monte Carlo 10^5^ samples in parameter space. Figure 5 illustrates the convergence of the Bayesian model evidence, the information entropy and the relative entropy estimates using GPE to the reference Monte Carlo solution during the Bayesian active learning. The RE-based active learning (blue line) convergences faster to the reference values than BME-based (red line) and RE-based (green line) active learning for all three indicators (BME, $D_{\mathrm{KL}}\left[p(\omega |D),p(\omega )\right]$ and $H\left[p(\omega |D)\right]$). Figure 5 aligns well with the results and discussion presented in Section 4.1.2.

**Figure 6:**
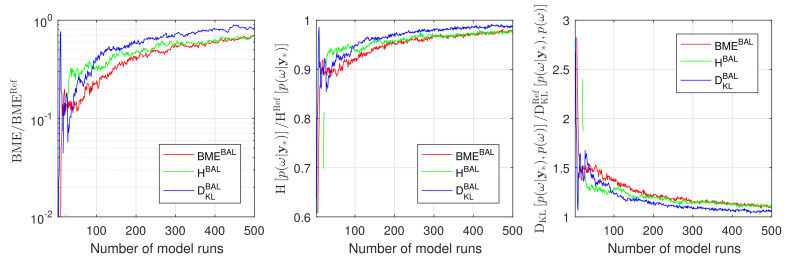
Convergence of Bayesian model evidence, Information entropy and Relative entropy estimates during active learning for Gaussian process emulator to the reference Monte Carlo solution for the 10D problem: BME-based active learning (red line), IE-based active learning (green line) and RE-based active learning (blue line).

Now, we will consider the full 10D setup of the problem (35) from Section 4.1.1. Similar to our discussion above, we will start the Bayesian active learning procedure with one training point only ($N_{T}=1$) corresponding to the mean value of uncertain parameters ω and we will employ again all three introduced strategies. Assessing the performance of the active learning procedures will also compute the BME values p(D), the RE value $D_{\mathrm{KL}}\left[p(\omega |D),p(\omega )\right]$ and the IE value $H\left[p(\omega |D)\right]$ based on the GPE surrogate. We will compare them against the reference values obtained from the plain MC technique on the original model with sample size of 10^5^. The results presented in Figure 6 confirm the anticipations from above and demonstrate a superior performance of RE-based active learning (blue line) in comparison to BME-based (red line) and IE-based active learning (green line). From the computational point of view, Figure 6 shows that the RE-based strategy already reaches an acceptable precision after approximately 200 model runs. This precision for the BME-based and the IE-based strategy can be reached, however, only after 500 model runs. It is worth mentioning that the current 10D setup is extremely challenging for GPE surrogates because of parameter dimensionality and its strong nonlinearity. From the current section, one can conclude that the relative entropy-based Bayesian active learning demonstrates a highly acceptable performance and seems to be the most suitable one for practical applications.

### 4.2. Bayesian Active Learning for Carbon Dioxide Benchmark Problem

#### 4.2.1. CO_2_ Benchmark Set up

We will consider a multi-phase flow problem in porous media, where carbon dioxide (CO_2_) is injected into a deep aquifer and then spreads in a geological formation. This yields a pressure build-up and a plume evolution. The CO_2_ injection into the subsurface could be a possible practice to mitigate the CO_2_ emission into the atmosphere. In this study, we use the deterministic model, provided by Köppel et al. [24], which is a reduced version of the model in a benchmark problem defined in the paper [85]. This reduction consists of a radial flow in the vicinity of the injection well, and made primarily due to the high computational demand of the original CO_2_ model. It is assumed that the fluid properties such as the density and the viscosity are constant, and all processes are isothermal. The CO_2_ and the brine build two separate and immiscible phases, and the mutual dissolution is neglected. Additionally, the formation is isotropically rigid and chemically inert, and capillary pressure is negligible. Overall, the considered CO_2_ benchmark problem is strongly nonlinear because the CO_2_ saturation spreaders as a strongly nonlinear front that could be challenging to capture via surrogates. For detailed information on the governing equations, the modeling assumption and the approaches, the reader is referred to the original publication [24].

Similar to [24], we consider the combined effects of three sources of uncertainty. We take into account the uncertainty of boundary conditions due to the injection rate, the uncertainty of parameters in the constitutive relations, introduced via uncertainty in the relative permeability definitions, and the uncertainty of material properties, i.e., the porosity of the geological layer. These three sources of uncertainty were introduced for the analysis in [24] using injection rate (IR), power theta (PT) and reservoir porosity (RP), i.e., $\omega =\left\{\mathrm{IR},\mathrm{PT},\mathrm{RP}\right\}$.

We consider the CO_2_ saturation to be the quantity of interest at a monitoring distance of 15 m from the injection well, measured each 10 days over a period of 100 days. We construct a scenario, in which the synthetic observed saturation values have been generated from the deterministic CO_2_ benchmark model itself, with the uncertain parameters to be set as $\omega_{Truth}=\left\{1.0e-04,0.2,0.3\right\}$. Additionally, we will assume that a measurement error of 0.02 ($\sigma_{D}=0.02$) exists for each synthetic observation data. Using the synthetic measurement data, we construct the reference solution conducting a Bayesian updating of the original CO_2_ benchmark model. Namely, reference values of Bayesian model evidence (${\mathrm{BME}}^{\mathrm{Ref}}$), the information entropy ($H^{\mathrm{Ref}}\left[p(\omega |D)\right]$) and the relative entropy $D_{\mathrm{KL}}^{\mathrm{Ref}}\left[p(\omega |D),p(\omega )\right]$ have been obtained based on 10^4^ Monte Carlo simulations using Equations (11), (15) and (19), correspondingly. Additionally, the posterior distribution of modeling parameters has been obtained via the same 10^4^ Monte Carlo simulations. One model run of the analyzed CO_2_ benchmark problem required approximately 3–7 min on a standard computer, depending strongly on the values of modeling parameters. In what follows, we present the results and analyze the performance of the three Bayesian active learning methods, introduced earlier in this paper, applied to the aforementioned CO_2_ benchmark set-up.

#### 4.2.2. Assessment of Information Arguments during Bayesian Active for CO_2_ Benchmarks

We start the Bayesian active learning process for the CO_2_ benchmark model with one training point only ($N_{T}=1$) using $\omega_{T}=E_{p(\omega )}\left(\omega \right)$. Similar to the previous applications in Section 4.1, we perform the Bayesian active learning procedure by using the BME-based, RE-based and IE-based strategies. Analogously, the performance of the active learning process is analyzed by comparing the BME values p(D), relative entropies $D_{\mathrm{KL}}\left[p(\omega |D),p(\omega )\right]$ and information entropies $H\left[p(\omega |D)\right]$ based on the GPE surrogates against their corresponding MC reference values.

**Figure 7:**
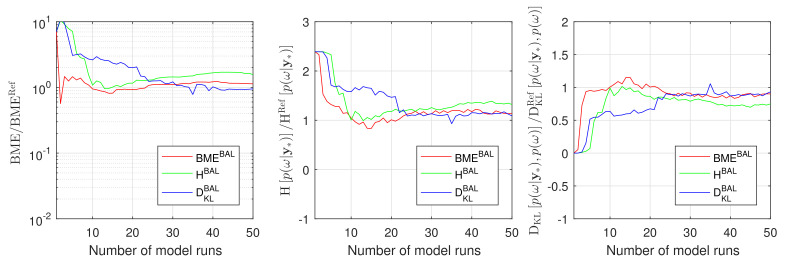
Convergence of Bayesian model evidence, Information entropy and Relative entropy estimates during active learning for Gaussian process emulator to the reference Monte Carlo solution for the CO_2_ Benchmark problem: BME-based active learning (red line), IE-based active learning (green line) and RE-based active learning (blue line).

Figure 7 illustrates the convergence of the BME value, the IE value and the RE value obtained during GPE-based Bayesian active learning against the reference Monte Carlo values. The results, presented in this figure, demonstrate that the RE-based (blue line) and the BME-based (red line) active learning shows again a superior performance compared to IE-based active learning (green line). Here, the RE-based strategy catches the reference BME values slightly better than the BME-based approach, and both approaches perform similarly well for other quantities of interest. The IE-based active learning demonstrates very similar behaviors to Section 4.1 and confirms the findings that have been reported for the 10D and its 2D reduction problems.

#### 4.2.3. Posterior Distribution of Modeling Parameters for CO_2_ Benchmarks

**Figure 8:**
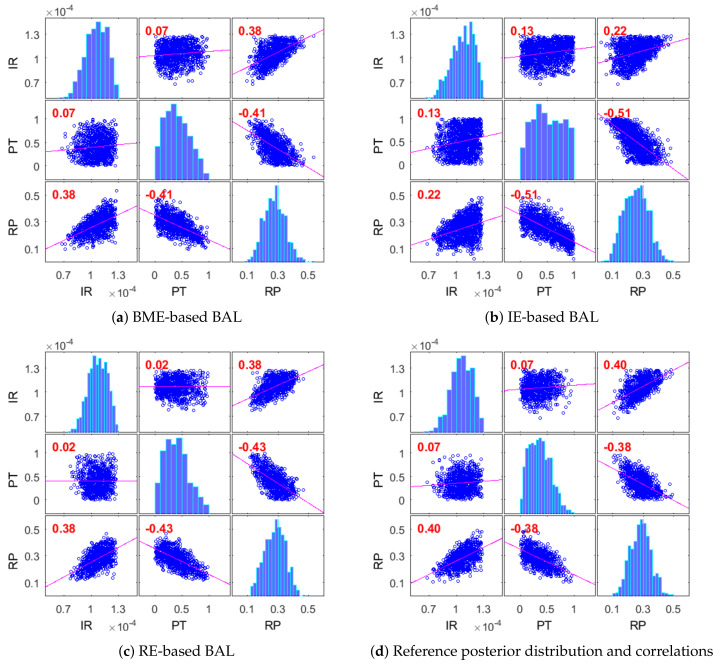
Posterior distributions and correlations of modeling parameters for the CO_2_ Benchmark problem after 100 active learning iterations: BME-based active learning (**a**), IE-based active learning (**b**), RE-based active learning (**c**) and reference Monte Carlo solution (**d**).

We will consider how good the introduced Bayesian active learning strategies can capture the posterior distribution of modeling parameters. To do so, we will illustrate the posterior distributions and correlations of modeling parameters for the CO_2_ Benchmark problem obtained after 50 active learning iterations. Figure 8 presents the results obtained using the BME-based active learning (Figure 8a), the IE-based active learning (Figure 8b) and the RE-based active learning (Figure 8c), and it compares them with the reference Monte Carlo solution (Figure 8d). The BME-based strategy in Figure 8a and the RE-based strategy in Figure 8c capture very well the posterior distributions of all analyzed parameters and their correlations in comparison to the MC reference solution. The information entropy-based strategy in Figure 8b captures acceptably the distributions and the correlations of the injection rate (IR) and the reservoir porosity (RP) parameters. However, it could not properly capture the distribution of the PT parameter controlling the relative permeability distribution. Figure 8b illustrates a very strong overestimation of high-value probabilities of this parameter. Very similar posterior distributions for all strategies have already been observed after 25 iterations of active learning, which corresponds to the convergence shown in Figure 7.

We have used 50 interactions for the GPE-based Bayesian active learning for the demonstrative purposes only. Apparently, such a high number of active learning iterations is unnecessary for practical applications. In the Bayesian context, a stabilization of the posterior distributions indicates convergence of the surrogate representation to the original model in the region of high posterior density. That property could be useful, especially once the reference solution cannot be constructed due to computation reasons, see [35,36]. The convergence of posterior distributions in Figure 8 aligns well with convergence of the information-theoretic indicators shown in Figure 7.

Overall, Section 4.2 indicates that the RE-based Bayesian active learning demonstrates a slightly superior performance over the BME-based strategy, and both strategies are superior to the IE-based approach. Obviously it is very easy to judge the performance of BAL once the reference solution is available. However, as in many practical cases, the reference solution is not available due to a very high computational demand and it is relevant to draw the conclusion without the reference solution. Apparently, all mentioned indicators such as BME value p(D), RE value $D_{\mathrm{KL}}\left[p(\omega |D),p(\omega )\right]$, and IE value $H\left[p(\omega |D)\right]$ reflect relevant information for a Bayesian inference. Therefore, the active learning procedure can be stopped once all such information-theoretic indicators stagnate and reach a plateau because the best possible surrogate representation of the original model have been (almost) reached.

### 4.3. Discussion

We use the link between Bayesian inference and information theory introduced in [40]. This means that we compute BME, RE and IE values while avoiding the criticized multi-Gaussian assumption. Instead, we perform prior-based sampling, i.e., Monte Carlo accompanied by rejecting sampling. Doing so, we consider that the main computations are related to running the original model and we assume feasibility of Monte Carlo sampling on the GPE surrogate.

Alternatively, any posterior-based sampling algorithm (e.g., MCMC) could be used during the Bayesian active learning procedure. However, when any posterior-based sampling algorithm is used, then the values for BME, RE and IE become quite rough approximations because relatively strong assumptions have to be taken. To provide cheap alternatives, various estimates of BME, RE and IE based on known criteria from information theory are now listed in Appendix A for the sake of completeness.

However, the estimates in Appendix A have to be used with care. According to [40], the harmonic mean estimate and the maximum-likelihood estimate for BME provide very unreliable results. Therefore, only rough guesses of the true BME value can be obtained from the maximum a posteriori estimate, Chib’s estimate [86], Bayesian information criterion [87] and the Akaike information criterion (with [83] and without second-order bias correction [88]) due to their strong assumptions. While estimates for BME, RE and IE based on the Kashyap information criterion [89] demonstrated unsatisfactory performance as well, we re-scaled them to a proper scale in [40]. However, the re-scaled Kashyap information criterion still includes unnecessary simplifications of the involved cross entropies. Among all these simplified estimates, the multivariate Gaussian posterior estimate [40] avoids the most unreasonable simplifications for posterior-based sampling and includes the least assumptions for estimating BME, RE and IE. The Gelfand and Dey approach [90] includes assumptions similar to the multivariate Gaussian, but provides slightly inferior results in the cases tested by us. Thus, for posterior-based sampling during Bayesian active learning, we suggested in our 2019 paper [40] to use the multivariate Gaussian estimate that includes least assumptions (Considering multivariate Gaussian distribution for active learning approaches focusing on GPE training on an underlying model only without considering measurement data is fully appropriate. It can be seen as Bayesian^2^ active learning and approximation signs in Equations (A25), (A26) and (A27) turn to equality containing no assumptions by definition of GPE.).

Finalizing the discussion, we would like to remark that straightforward applications of the suggested Bayesian active learning strategies (or even already existing approaches) to GPE representations could be computationally very demanding once the problem dimensionality increases. This is mainly caused by the structure of GPE surrogates that is represented via localized kernels. These localized kernels require a lot of training for high-dimensional cases. Increasing the amount of measurement data will help to localize the relevant spots better. However, for high-dimensional problems, the structure of the surrogate should be constructed adaptively and sparse representations will be very beneficial. Alternatively, a preliminary sensitivity analysis (see e.g., [91,92]) could be conducted to partially overcome the problem of dimensionality.

## 5. Summary and Conclusions

The current paper deals with Gaussian process emulator that replicates a computational demanding physical model and honors the available observation data establishing fully Bayesian^3^ active learning framework. We elaborate the connection between Bayesian inference and information theory and offer a fully Bayesian view on a Gaussian process emulator through a Bayesian inference accompanied by a Bayesian active learning.

The paper employs the fundamental properties of Gaussian process emulator and introduces, in Section 3, three Bayesian active learning strategies. These strategies adaptively identify training sets, for which the full-complexity model must be evaluated. The first Bayesian active learning strategy, relying on Bayesian model evidence, indicates the quality of representation against the available measurements data. The second Bayesian active learning strategy, based on the relative entropy, seeks a relative information gain. The third Bayesian active learning strategy, based on information entropy, considers the expected missing information. The introduced strategies improve the Gaussian process emulator-based surrogate representation of a full-complexity physical model in the region of high posterior density. We employ the information-theoretic arguments to incorporate adaptively the measurements data. We emphasize in the paper that the information entropy and the relative entropy can be computed avoiding any assumption or unnecessary multidimensional integration.

We illustrate the performance of the suggested Bayesian active learning strategies using an analytical example and a carbon dioxide benchmark. Section 4 shows how the suggested approaches capture the likelihood values during an active learning procedure. We also show a visual comparison with the reference Monte Carlo solution for a 2D reduction of the 10D problem. We demonstrate rigorous evidence of convergence against the reference Monte Carlo values for the Bayesian model evidence, the information entropy and the relative entropy obtained via the three Bayesian active learning strategies. Additionally, Section 4 shows the evidence of convergence for the carbon dioxide benchmark problem against the reference solution for all proposed Bayesian active learning strategies. We also illustrate how the suggested Bayesian active learning strategies manage to quantify the post-calibration uncertainty in comparison to available Monte Carlo reference solutions.

Overall, we conclude that the introduced Bayesian active learning strategies for Gaussian process emulators could be very helpful for applied tasks where underlying full-complexity models are computationally very expensive. Moreover, the employed information-theoretic indicators can be used as stop criteria for Bayesian active learning once a reference solution is not available due to a very high computational demand. Our analysis indicates that the Bayesian model evidence-based and the relative entropy-based strategy demonstrate more reliable results in comparison to the information entropy-based strategy, which could be misleading. Additionally, the relative entropy-based strategy demonstrates a superior performance relative to the Bayesian model evidence-based strategy and seems to provide very sensitive arguments for the active learning. 

## Figures and Tables

**Figure A1:**
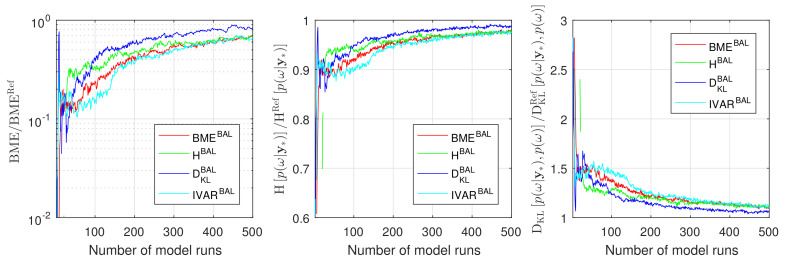
Convergence of Bayesian model evidence, Information entropy and Relative entropy estimates during active learning for Gaussian process emulator to the reference Monte Carlo solution for the 10D problem: BME-based active learning (red line), IE-based active learning (green line), RE-based active learning (blue line) and IVAR-based active learning (teal line).

## Appendix A. List of Approximative Active Learning Strategies

The current section briefly summarizes the alternative learning strategies that are based on certain assumptions. The approximates for BME, RE and IE values have been adopted from the paper [40] to Bayesian active learning introduced in Section 3 and we refer the reader to the original work for the complete details. All estimates mentioned here are less powerful than the strategies introduced in Section 3 due to their definitions. The most promising approximates are based as well on the multivariate Gaussian assumptions of the posterior $p_{\omega_{E}}^{BAL}\left(\omega |D\right)$ in Equation (21).

As it has been mentioned already in Section 1, learning functions for GPE training in Bayesian parameter inference can focus on the posterior mean and variance of model parameters obtained via assimilation of available measurement data. Doing so, we have adopted the idea of minimizing the integrated posterior variance (IVAR) of the GPE [77] to perform an active learning in the context of Bayesian assimilation of available data. Figure A1 illustrates convergence of active learning based on the IVAR strategy for the 10D setup discussed in Section 4.1.3. The IVAR-based active learning strategy (teal line) shows promising results while relying on low-order moments only. However, all three strategies introduced in the current paper demonstrate faster convergence, capturing the relevant spots at low computational budget and, as it has been already pointed out in Section 4, the RE-based active learning strategy demonstrates superior results. The reason is that the posterior distribution $p_{\omega_{E}}^{BAL}\left(\omega |D\right)$ resulting from Bayesian assimilation of measurement data is typically not multivariate Gaussian and corresponding assumptions slow down the active learning procedure.

### Appendix A.1. Maximum a Posteriori Estimates

The BME, RE and IE values could be approximated as follows using the maximum a posteriori (MAP) estimates [40]:

(A1) $$\ln{\mathrm{BME}}_{\mathrm{MAP}}^{\mathrm{BAL}}\approx E_{p_{\omega_{E}}^{BAL}\left(S|D\right)}\left(\ln\left[p_{\omega_{E}}^{BAL}\left(D|S\right)\right]\right)+E_{p_{\omega_{E}}^{BAL}\left(S|D\right)}\left(\ln\left[N_{\omega_{E}}\left(\mu_{S},\sigma_{S}\right)\right]\right)-\ln\left[p_{\omega_{E}}^{BAL}\left(S_{MAP}|D\right)\right],$$

(A2) $$D_{{\mathrm{KL}}_{\mathrm{MAP}}}^{\mathrm{BAL}}\left[p_{\omega_{E}}^{BAL}\left(S|D\right),N_{\omega_{E}}\left(\mu_{S},\sigma_{S}\right)\right]\approx -E_{p_{\omega_{E}}^{BAL}\left(S|D\right)}\left(\ln\left[N_{\omega_{E}}\left(\mu_{S},\sigma_{S}\right)\right]\right)+\ln\left[p_{\omega_{E}}^{BAL}\left(S_{MAP}|D\right)\right],$$

(A3) $$H_{\mathrm{MAP}}^{\mathrm{BAL}}\left[p_{\omega_{E}}^{BAL}\left(S|D\right)\right]\approx -\ln\left[p_{\omega_{E}}^{BAL}\left(S_{MAP}|D\right)\right],$$

where $S_{MAP}$ is a maximum a posteriori response of the surrogate S.

#### Appendix A.1.1. Chib’s Estimates

Following the idea of Chib [86], a single point estimate could be used in the following way [40]:

(A4) $$\ln{\mathrm{BME}}_{\mathrm{CHIB}}^{\mathrm{BAL}}\approx \ln\left[p_{\omega_{E}}^{BAL}\left(D|S_{MAP}\right)\right]+\ln\left[N_{\omega_{E}}\left(\mu_{S},\sigma_{S}\right)\left(S_{MAP}\right)\right]-\ln\left[p_{\omega_{E}}^{BAL}\left(S_{MAP}|D\right)\right].$$

(A5) $$D_{{\mathrm{KL}}_{\mathrm{CHIB}}}^{\mathrm{BAL}}\left[p_{\omega_{E}}^{BAL}\left(S|D\right),N_{\omega_{E}}\left(\mu_{S},\sigma_{S}\right)\right]\approx -\ln\left[N_{\omega_{E}}\left(\mu_{S},\sigma_{S}\right)\left(S_{MAP}\right)\right]+\ln\left[p_{\omega_{E}}^{BAL}\left(S_{MAP}|D\right)\right],$$

(A6) $$H_{\mathrm{CHIB}}^{\mathrm{BAL}}\left[p_{\omega_{E}}^{BAL}\left(S|D\right)\right]\approx -\ln\left[p_{\omega_{E}}^{BAL}\left(S_{MAP}|D\right)\right].$$

#### Appendix A.1.2. Estimates via Akaike Information Criterion

The MAP approximation could be extended while employing the Akaike information criterion (AIC) [83] as follows [40]:

(A7) $$\ln{\mathrm{BME}}_{\mathrm{AIC}}^{\mathrm{BAL}}\approx E_{p_{\omega_{E}}^{BAL}\left(S|D\right)}\left(\ln\left[p_{\omega_{E}}^{BAL}\left(D|S\right)\right]\right)+E_{p_{\omega_{E}}^{BAL}\left(S|D\right)}\left(\ln\left[N_{\omega_{E}}\left(\mu_{S},\sigma_{S}\right)\right]\right)-\frac{1}{n}\ln\left[p_{\omega_{E}}^{BAL}\left(S_{MAP}|D\right)\right]+1,$$

(A8) $$D_{{\mathrm{KL}}_{\mathrm{AIC}}}^{\mathrm{BAL}}\left[p_{\omega_{E}}^{BAL}\left(S|D\right),N_{\omega_{E}}\left(\mu_{S},\sigma_{S}\right)\right]\approx -E_{p_{\omega_{E}}^{BAL}\left(S|D\right)}\left(\ln\left[N_{\omega_{E}}\left(\mu_{S},\sigma_{S}\right)\right]\right)+\frac{1}{n}\ln\left[p_{\omega_{E}}^{BAL}\left(S_{MAP}|D\right)\right]-1,$$

(A9) $$H_{\mathrm{AIC}}^{\mathrm{BAL}}\left[p_{\omega_{E}}^{BAL}\left(S|D\right)\right]\approx -\frac{1}{n}\ln\left[p_{\omega_{E}}^{BAL}\left(S_{MAP}|D\right)\right]+1.$$

#### Appendix A.1.3. Estimates via Second-Order bias Correction for Akaike Information Criterion

Second-order bias correction [88] for a limited sample size *s* (length of vector D) extends the Akaike information criterion to the following from [40]:

(A10) $$\begin{array}{cc} \ln{\mathrm{BME}}_{\mathrm{AICc}}^{\mathrm{BAL}}\approx & E_{p_{\omega_{E}}^{BAL}\left(S|D\right)}\left(\ln\left[p_{\omega_{E}}^{BAL}\left(D|S\right)\right]\right)+E_{p_{\omega_{E}}^{BAL}\left(S|D\right)}\left(\ln\left[N_{\omega_{E}}\left(\mu_{S},\sigma_{S}\right)\right]\right)-\\ \; & -\frac{1}{n}\ln\left[p_{\omega_{E}}^{BAL}\left(S_{MAP}|D\right)\right]+\frac{s}{s-n-1}. \end{array}$$

(A11) $$\begin{array}{cc} D_{{\mathrm{KL}}_{\mathrm{AICc}}}^{\mathrm{BAL}}\left[p_{\omega_{E}}^{BAL}\left(S|D\right),N_{\omega_{E}}\left(\mu_{S},\sigma_{S}\right)\right]\approx & -E_{p_{\omega_{E}}^{BAL}\left(S|D\right)}\left(\ln\left[N_{\omega_{E}}\left(\mu_{S},\sigma_{S}\right)\right]\right)+\\ \; & +\frac{1}{n}\ln\left[p_{\omega_{E}}^{BAL}\left(S_{MAP}|D\right)\right]-\frac{s}{s-n-1}, \end{array}$$

(A12) $$H_{\mathrm{AICc}}^{\mathrm{BAL}}\left[p_{\omega_{E}}^{BAL}\left(S|D\right)\right]\approx -\frac{1}{n}\ln\left[p_{\omega_{E}}^{BAL}\left(S_{MAP}|D\right)\right]+\frac{s}{s-n-1}.$$

#### Appendix A.1.4. Estimates via Bayesian Information Criterion

Employing the Bayesian information criterion (also known as Schwarz information criterion) introduced by Schwarz [87] leads to the following estimates [40]:

(A13) $$\ln{\mathrm{BME}}_{\mathrm{BIC}}^{\mathrm{BAL}}\approx \ln\left[p_{\omega_{E}}^{BAL}\left(S_{MLE}|D\right)\right]+\frac{n}{2}\ln s,$$

(A14) $$D_{{\mathrm{KL}}_{\mathrm{BIC}}}^{\mathrm{BAL}}\left[p_{\omega_{E}}^{BAL}\left(S|D\right),N_{\omega_{E}}\left(\mu_{S},\sigma_{S}\right)\right]\approx \ln\left[p_{\omega_{E}}^{BAL}\left(D|S_{MLE}\right)\right]-\ln\left[p_{\omega_{E}}^{BAL}\left(S_{MLE}|D\right)\right]-\frac{n}{2}\ln s,$$

(A15) $$H_{\mathrm{BIC}}^{\mathrm{BAL}}\left[p_{\omega_{E}}^{BAL}\left(S|D\right)\right]\approx \ln\left[p_{\omega_{E}}^{BAL}\left(S_{MLE}|D\right)\right]-\ln\left[N_{\omega_{E}}\left(\mu_{S},\sigma_{S}\right)\left(S_{MLE}\right)\right]-\ln\left[p_{\omega_{E}}^{BAL}\left(D|S_{MLE}\right)\right]+\frac{n}{2}\ln s,$$

where $S_{MLE}$ is a maximum a posteriori response of the surrogate S.

#### Appendix A.1.5. Estimates via Kashyap Information Criterion

Approximations based on the Kashyap Information Criterion [89] offer the following estimates [40]:

(A16) $$\ln{\mathrm{BME}}_{\mathrm{KIC}}^{\mathrm{BAL}}\approx \ln\left[p_{\omega_{E}}^{BAL}\left(S_{MAP}|D\right)\right]+\ln\left[N_{\omega_{E}}\left(\mu_{S},\sigma_{S}\right)\left(S_{MAP}\right)\right]+\frac{1}{2}\ln\left[{\left(2\pi \right)}^{n}\left|C\right|\right].$$

(A17) $$D_{{\mathrm{KL}}_{\mathrm{KIC}}}^{\mathrm{BAL}}\left[p_{\omega_{E}}^{BAL}\left(S|D\right),N_{\omega_{E}}\left(\mu_{S},\sigma_{S}\right)\right]\approx -\ln\left[N_{\omega_{E}}\left(\mu_{S},\sigma_{S}\right)\left(S_{MAP}\right)\right]-\frac{1}{2}\ln\left[{\left(2\pi \right)}^{n}\left|C\right|\right],$$

(A18) $$H_{\mathrm{KIC}}^{\mathrm{BAL}}\left[p_{\omega_{E}}^{BAL}\left(S|D\right)\right]\approx \frac{1}{2}\ln\left[{\left(2\pi \right)}^{n}\left|C\right|\right].$$

#### Appendix A.1.6. Estimates via Re-Scaled Kashyap Information Criterion

The re-scaled correction of Kashyap Information Criterion according to paper [40] leads to the following:

(A19) $$\ln{\mathrm{BME}}_{\mathrm{KICr}}^{\mathrm{BAL}}\approx \ln\left[p_{\omega_{E}}^{BAL}\left(S_{MAP}|D\right)\right]+\ln\left[N_{\omega_{E}}\left(\mu_{S},\sigma_{S}\right)\left(S_{MAP}\right)\right]+\frac{1}{2}\ln\left[{\left(2\pi e\right)}^{n}\left|C\right|\right].$$

(A20) $$D_{{\mathrm{KL}}_{\mathrm{KICr}}}^{\mathrm{BAL}}\left[p_{\omega_{E}}^{BAL}\left(S|D\right),N_{\omega_{E}}\left(\mu_{S},\sigma_{S}\right)\right]\approx -\ln\left[N_{\omega_{E}}\left(\mu_{S},\sigma_{S}\right)\left(S_{MAP}\right)\right]-\frac{1}{2}\ln\left[{\left(2\pi e\right)}^{n}\left|C\right|\right],$$

(A21) $$H_{\mathrm{KICr}}^{\mathrm{BAL}}\left[p_{\omega_{E}}^{BAL}\left(S|D\right)\right]\approx \frac{1}{2}\ln\left[{\left(2\pi e\right)}^{n}\left|C\right|\right],$$

where C is the posterior (co)variance matrix.

#### Appendix A.1.7. Estimates via Gelfand and Dey Sampling

Following the idea of Gelfand and Dey [90], an importance sampling with density τ(S) leads to the following approximates [40]:

(A22) $$\ln{\mathrm{BME}}_{\mathrm{GD}}^{\mathrm{BAL}}\approx \ln E_{p_{\omega_{E}}^{BAL}\left(S|D\right)}^{-1}\left(\frac{\tau (S)}{p_{\omega_{E}}^{BAL}\left(D|S\right)N_{\omega_{E}}\left(\mu_{S},\sigma_{S}\right)}\right),$$

(A23) $$\begin{array}{cc} D_{{\mathrm{KL}}_{\mathrm{GD}}}^{\mathrm{BAL}}\left[p_{\omega_{E}}^{BAL}\left(S|D\right),N_{\omega_{E}}\left(\mu_{S},\sigma_{S}\right)\right]\approx & E_{p_{\omega_{E}}^{BAL}\left(S|D\right)}\left(\ln\left[p_{\omega_{E}}^{BAL}\left(D|S\right)\right]\right)-\\ \; & -\ln E_{p_{\omega_{E}}^{BAL}\left(S|D\right)}^{-1}\left(\frac{\tau (S)}{p_{\omega_{E}}^{BAL}\left(D|S\right)N_{\omega_{E}}\left(\mu_{S},\sigma_{S}\right)}\right), \end{array}$$

(A24) $$\begin{array}{cc} H_{\mathrm{GD}}^{\mathrm{BAL}}\left[p_{\omega_{E}}^{BAL}\left(S|D\right)\right]\approx & -E_{p_{\omega_{E}}^{BAL}\left(S|D\right)}\left(\ln\left[p_{\omega_{E}}^{BAL}\left(D|S\right)\right]\right)-E_{p_{\omega_{E}}^{BAL}\left(S|D\right)}\left(\ln\left[N_{\omega_{E}}\left(\mu_{S},\sigma_{S}\right)\right]\right)+\\ \; & +\ln E_{p_{\omega_{E}}^{BAL}\left(S|D\right)}^{-1}\left(\frac{\tau (S)}{p_{\omega_{E}}^{BAL}\left(D|S\right)N_{\omega_{E}}\left(\mu_{S},\sigma_{S}\right)}\right), \end{array}$$

where the importance sampling density τ(S) is often assumed to be multivariate Gaussian or *t*-distributed.

#### Appendix A.1.8. Multivariate Gaussian Estimates

Assumption on multivariate Gaussian (MG) distribution [93,94] of the posterior distribution $p_{\omega_{E}}^{BAL}\left(S|D\right)$ leads to the following approximates [40]:

(A25) $$\ln{\mathrm{BME}}_{\mathrm{MG}}^{\mathrm{BAL}}\approx E_{p_{\omega_{E}}^{BAL}\left(S|D\right)}\left(\ln\left[p_{\omega_{E}}^{BAL}\left(D|S\right)\right]\right)+E_{p_{\omega_{E}}^{BAL}\left(S|D\right)}\left(\ln\left[N_{\omega_{E}}\left(\mu_{S},\sigma_{S}\right)\right]\right)+\frac{1}{2}\ln\left[{\left(2\pi e\right)}^{n}\left|C\right|\right],$$

(A26) $$D_{\mathrm{KL}}\left[p_{\omega_{E}}^{BAL}\left(S|D\right),N_{\omega_{E}}\left(\mu_{S},\sigma_{S}\right)\right]=-E_{p_{\omega_{E}}^{BAL}\left(\omega |D\right)}\left(\ln\left[N_{\omega_{E}}\left(\mu_{S},\sigma_{S}\right)\right]\right)-\frac{1}{2}\ln\left[{\left(2\pi e\right)}^{n}\left|C\right|\right],$$

(A27) $$H\left[p_{\omega_{E}}^{BAL}\left(\omega |D\right)\right]\approx \frac{1}{2}\ln\left[{\left(2\pi e\right)}^{n}\left|C\right|\right].$$
